# Efficacy of electroacupuncture in improving postoperative ileus in patients receiving colorectal surgery: a systematic review and meta-analysis

**DOI:** 10.1097/JS9.0000000000000848

**Published:** 2023-11-02

**Authors:** Hsiao-Tien Chen, Kuo-Chuan Hung, Yen-Ta Huang, Jheng-Yan Wu, Chung-Hsi Hsing, Chien-Ming Lin, I-Wen Chen, Cheuk-Kwan Sun

**Affiliations:** a Department of Chinese Medicine Chi Mei Medical Center, Tainan; b Department of Anesthesiology Chi Mei Medical Center, Liouying, Tainan; c Department of Anesthesiology, Chi Mei Medical Center, Tainan; d Department of Medical Research Chi Mei Medical Center, Tainan; e Department of Nutrition, Chi Mei Medical Center Tainan; f Department of Surgery, National Cheng Kung University Hospital, College of Medicine, National Cheng Kung University, Tainan; g School of Medicine, College of Medicine, National Sun Yat-sen University, Kaohsiung; h Department of Emergency Medicine, E-Da Dachang Hospital, I-Shou University, Kaohsiung; i School of Medicine for International Students, College of Medicine, I-Shou University, Kaohsiung, Taiwan

**Keywords:** acupuncture, colorectal surgery, electroacupuncture, gastrointestinal function, postoperative ileus, recovery

## Abstract

**Background:**

This meta-analysis aimed to evaluate the efficacy and safety of electroacupuncture (EA) in improving postoperative ileus after colorectal surgery.

**Methods:**

Electronic databases (e.g. Medline) were screened to identify randomized controlled trials that focused on the association between EA and postoperative ileus. Time to first flatus served as the primary outcome, while the secondary outcomes included time required for the recovery of other gastrointestinal functions (e.g. bowel sound recovery), time to tolerability of liquid/solid food, postoperative pain scores, risk of overall complications, and hospital length of stay.

**Results:**

Our meta-analysis focusing on 16 studies with a total of 1562 patients demonstrated positive associations of EA with shorter times to the first flatus [mean difference (MD): −10.1 h, *P*<0.00001, *n*=1562], first defecation (MD: −11.77 h, *P*<0.00001, *n*=1231), bowel sound recovery (MD: −10.76 h, *P*<0.00001, *n*=670), tolerability of liquid (MD: −16.44 h, *P*=0.0002, *n*=243), and solid food (MD: −17.21 h, *P*=0.005, *n*=582) than those who received standard care. The use of EA was also correlated with a lower risk of overall complications (risk ratio:0.71, *P*=0.04, *n*=1011), shorter hospital length of stay (MD: −1.22 days, *P*=0.0001, *n*=988), and a lower pain score on postoperative days two (standardized MD: −0.87, *P*=0.009, *n*=665) and three (standardized MD: −0.45, *P*<0.00001, *n*=795), without a difference in time to first ambulation.

**Conclusion:**

Our findings showed an association between EA and enhanced gastrointestinal functional recovery and reduced pain severity following colorectal surgery, highlighting the potential benefits of incorporating EA into perioperative care to enhance recovery outcomes in this setting.

## Introduction

Highlights

- The efficacy of electroacupuncture (EA) for postoperative ileus was investigated.
- Sixteen trials with 1578 patients receiving colorectal surgery were meta-analyzed.
- EA was linked to shorter times to first flatus/defecation and bowel sound recovery.
- EA correlated with reduced time to food tolerance and pain score on days 2 and 3.
- EA was associated with a lower risk of complications and a shorter hospital stay.

Colorectal cancer (CRC) is a highly prevalent malignancy worldwide, accounting for 9.4% of all cancer incidences in men and 10.1% in women^1^. More importantly, the total number of fatalities from rectal and colon cancers is speculated to increase by 60 and 71.5%, respectively^2^. Although surgical treatment remains the primary curative approach, various operation-related factors, including anesthesia, surgical trauma, inflammatory reactions, and the use of opioids, can impair postoperative gastrointestinal motility^3^. In particular, 10–30% of patients undergoing colorectal surgery reported the occurrence of postoperative ileus (POI)^3,4^ characterized by a range of symptoms, including abdominal distension, pain, delayed passage of gas and stool, nausea, vomiting, and an inability to tolerate oral intake^5^. Despite the typical resolution of POI within 5 days following open abdominal surgery and within three days following laparoscopic surgery^6^, its occasional presentation as a prolonged and recurrent disorder may lead to severe morbidities including electrolyte imbalances, dehydration, and sepsis^7^. Given the global burden of CRC and the high incidence of POI following surgical interventions, appropriate implementation of preventive strategies, close monitoring, and timely interventions for POI are crucial for minimizing associated morbidities and improving patient recovery.

Acupuncture, a traditional Chinese medicine technique originating in East Asia that has been practiced for centuries, involves the stimulation of specific acupoints to alleviate various gastrointestinal symptoms^8^. In recent years, acupuncture has gained popularity as a nonpharmacological and minimally invasive therapeutic option for various gastrointestinal disorders, including abdominal distension, pain, constipation, nausea, and vomiting^9–11^. Several meta-analyses have shown the effectiveness of acupuncture and related techniques, such as electroacupuncture (EA) or transcutaneous acupoint electrical stimulation (TEAS), in improving POI among patients undergoing abdominal surgery^12–14^. However, the significant heterogeneity within these meta-analyses raises concerns regarding the robustness and generalizability of the evidence generated.

EA is a contemporary clinical approach that integrates traditional acupuncture techniques with the precise application of stabilized electrical stimulation, enabling objective, and quantifiable adjustments of stimulation frequency and intensity^15^. This unique characteristic has garnered considerable attention, focusing on EA as a potential therapeutic intervention for patients with CRC who develop POI^16^. Therefore, the primary objective of this systematic review and meta-analysis was to examine the reliability and validity of currently available evidence supporting the use of EA to improve POI among patients with CRC.

## Methods

The study protocol can be accessed from the PROSPERO International prospective register of the systematic review database. This study adhered to the Preferred Reporting Items for Systematic Reviews and Meta-Analyses Statement (PRISMA, Supplemental Digital Contents 3 and 4, http://links.lww.com/JS9/B234, http://links.lww.com/JS9/B235)^17^ and AMSTAR (Supplemental Digital Content 2, http://links.lww.com/JS9/B233) (Assessing the methodological quality of systematic reviews)^18^ guidelines when reporting its findings.

### Search strategies and data sources

A comprehensive literature search was conducted to identify randomized controlled trials (RCTs) that investigated the effectiveness of EA in improving postoperative gastrointestinal motility disorders in patients undergoing colorectal surgery. The search encompassed major databases, including PubMed, MEDLINE, Embase, and the Cochrane CENTRAL register of controlled trials, spanning from their inception dates until 8 June 2023. A search of the ClinicalTrials. gov database was also performed to ensure the inclusion of ongoing or unpublished studies. The search strategy employed a combination of keywords and medical subject headings (e.g. MeSH terms in Medline), including the following keywords: (ʻAcupunctureʼ or ʻElectroacupuncture therapyʼ or ʻElectroacupunctureʼ or ʻElectro acupuncturingʼ or ʻLaser acupunctureʼ or ʻElectro needle acupunctureʼ or ʻElectro auricular acupunctureʼ) and (ʻcolorectal cancerʼ or ʻcolorectal carcinomaʼ or ʻcolon adenocarcinomaʼ or ʻcolorectal tumorʼ or ʻcolonic neoplasmʼ or ʻrectal cancerʼ or ʻrectosigmoid tumorʼ or ʻsigmoid colon tumorʼ) and (ʻpostoperative ileusʼ or ʻpostoperative bowel dysfunctionʼ or ʻpostoperative gastrointestinal dysfunctionʼ or ʻparalytic ileusʼ). Furthermore, to prevent the omission of relevant studies, Google Scholar, the China National Knowledge Infrastructure (CNKI) database, Chongqing VIP Database (CQVIP), Chinese Biomedical Literature Database (CBM), and the Wanfang Database were also searched. To identify additional studies, the reference lists of the published systematic reviews and included RCTs were screened. No restrictions were placed on the language, sample size, publication date, or country of origin.

### Inclusion criteria and study selection

The following PICOS criteria were used to screen eligible trials: (P) participants: adults receiving colorectal surgery regardless of the surgical techniques (e.g. laparoscopy or laparotomy); (I) interventions: the use of EA with or without other adjunct therapies to improve POI; (C) comparisons: sham acupuncture or standard care; (O) Outcomes: POI-related outcomes were available [for example, time to first flatus (TFF)]; and (S) study design: only RCTs were included.

The following criteria were used to exclude ineligible studies: (a) studies that included patients receiving both colorectal and other gastrointestinal surgeries (e.g. gastrectomy); (b) acupuncture-related therapy was also used in the control group; (c) studies that focused on manual acupuncture or TEAS; and (d) full-text or POI-related outcomes were unavailable.

Following the removal of duplicate records, potentially eligible studies underwent a screening process conducted by two independent reviewers who evaluated the titles and abstracts. Full-text articles were obtained and reviewed by the same reviewers to determine the inclusion criteria. In cases of disagreement, a third reviewer was consulted to facilitate discussion and reach consensus.

### Data extraction

The data extraction process was conducted independently by two reviewers who extracted relevant information using a standardized data extraction form. The extracted data included the first author’s name, publication year, baseline characteristics of participants (e.g. age), size of the study population, surgical techniques (e.g. laparoscopy or laparotomy), details of the EA procedure, interventions in controls (e.g. sham acupuncture or standard care), and country. Outcomes, including time to first flatus (TFF), time to bowel sound recovery (TBSR), time to first defecation (TFD), time to liquid/semiliquid/solid food intake, postoperative pain score, postoperative overall complications, and hospital length of stay (LOS) were also retrieved. In cases of divergent opinions, a third reviewer made a final decision to resolve any discrepancies.

### Outcomes and definitions

TFF, defined in accordance with the individual studies, was the primary endpoint of the current meta-analysis. Additionally, several secondary outcomes were evaluated, including TBSR, TFD, time to initiate intake of liquid or solid food, postoperative pain scores, postoperative complications, and hospital LOS. In cases where pain scores were measured at multiple time points, data were gathered from the first three postoperative days.

### Quality of included studies

The methodological quality of the included trials was assessed by two reviewers using the Cochrane Risk of bias (ROB) tool. The RoB2 framework encompasses diverse domains crucial for the examination of potential biases, namely, the randomization process, deviations from intended interventions, missing outcome data, measurement of outcomes, selection of reported results, and overall bias. Any disagreements between the two reviewers were discussed, and if unresolved, a third reviewer was involved in the discussion until consensus was reached.

### Certainty of evidence

The Grading of Recommendations Assessment, Development, and Evaluation (GRADE) approach was employed to evaluate the overall quality of the evidence in this study. The GRADE guidelines examine five domains: risk of bias, inconsistency, indirectness, imprecision, and potential publication bias. These domains collectively contribute to the assessment of evidence quality and are classified into four levels: high, moderate, low, and very low. To ensure objectivity and reliability, two researchers independently conducted the evaluation, whereas a third researcher reviewed the assessment process. Any discrepancies or disagreements were resolved through thorough discussion and consultation with professional specialists.

### Data analysis

The meta-analysis was conducted using the Review Manager (RevMan) version 5.4.1 software. We presented categorized variables as risk ratios (RRs) with 95% CIs. Continuous variables were analyzed using the mean difference (MD) with a 95% CI, when the units were consistent. In cases where the units varied, standardized mean difference (SMD) was employed. The effect size of SMD was interpreted as follows: a value of less than 0.2 represented a small effect, 0.5 indicated a moderate effect, and a value of 0.8 or greater signified a large effect. To prevent overlap in sample size assessment in studies with more than two intervention/control arms, a strategy was employed in which participants in the intervention and control groups were divided into separate subgroups for comparison with their counterparts in the corresponding specific intervention/control arms, as described in a previous study^19^. This approach ensured that means and SD were preserved for continuous variables, whereas for categorical outcomes, both the event number and total number of participants were divided. If the *I*^2^ value exceeded 50%, the heterogeneity was considered significant. Given the variation in clinical settings, a random effects model was used. To investigate the sources of heterogeneity, subgroup analyses based on the type of surgery (laparoscopy vs. laparotomy), type of control group (sham-EA vs. standard care), position of acupoint (ST36-based EA vs. others), and time of EA (preoperative vs. postoperative) were conducted to analyze the primary outcome. A sensitivity analysis was performed by sequentially excluding each RCT to assess the robustness of the results. When at least 10 trials or data sets were included, a funnel plot was used to detect potential publication bias when at least ten trials or datasets were included. Statistical significance was set at a *P*-value of less than 0.05.

## Results

### Search results

**Figure 1:**
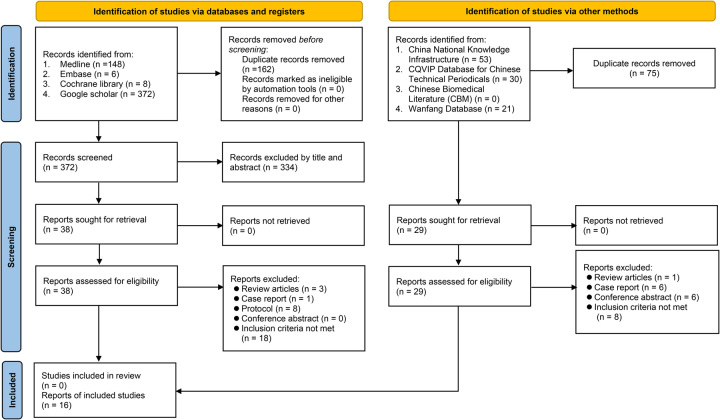
Flowchart for inclusion and exclusion of studies.

Of the initial 534 records obtained from the four electronic databases, 162 were eliminated because of duplications. Among the remaining 372 records, 334 were considered ineligible based on the assessment of their titles and abstracts. Subsequently, following a thorough full-text screening of the remaining 38 trials, an additional 30 reports were excluded, resulting in eight RCTs that met the eligibility criteria for inclusion in the present study (Fig. 1). Furthermore, during the examination of other databases, such as CNKI, an additional eight RCTs were identified. Overall, a total of 16 RCTs (*n*=1562 patients) conducted in China published between 2013 and 2023 were included in the analysis^16,20–34^.

### Characteristics of studies

The characteristics of the studies included in this meta-analysis are summarized in Table 1. Baseline patient characteristics, including age and sex, were available for 15 studies. Participants in the EA group were aged 49–67, while those in the control group were aged 51–69. The proportion ranged from 47.5 to 64.6%. However, one study did not provide any relevant information^32^. Of the 16 eligible RCTs, seven focused exclusively on laparoscopic colorectal surgery^16,20,21,25,31,33,35^, while four only recruited adults undergoing open colorectal surgery^23,28,30,34^. Two trials included patients who underwent either laparoscopic or open colorectal surgery^27,29^. Additionally, three trials did not provide specific details on the type of surgery conducted^22,24,32^. The sample size in the EA group ranged from 19 to 125 participants, whereas it ranged from 20 to 124 participants in the control group. Of the 16 trials, six employed a triple-arm study design^21,23,25,27,32,33^, while two utilized a four-arm design^28,30^.

**Table 1:** Characteristics of studies (*n*=16).

						Groups			
References	Age (years)^a^	Male (%)	Type of surgery	Sample size^a^ (*n*=1562)	Intervention time (min)	Intervention	Control	Outcomes	Country
Deng *et al*.^16^	59 vs. 62	58.3	LCS	30 vs. 30	30	EA	Standard care	1,2,4,5,7,10,11	China
Gong *et al*.^20^	65 vs. 66	52.5	LCS	62 vs. 58	30	EA	Standard care	1,2,4,5,11	China
Li *et al*.^21^	61/61 vs. 61^b^	47.5	LCS	80 vs. 40	30	EA	Sham-EA	1,2,3	China
Long *et al*.^22^	64 vs. 62	53.3	NA	30 vs. 30	30	EA	Standard care	1,2,3,4,5,11	China
Mai *et al*.^23^	50/52 vs. 51^b^	60	OCS	40 vs. 20	30	EA	Standard care	1,2,3	China
Meng *et al*.^24^	53.7	55	NA	35 vs. 40	20	EA	Standard care	1,3,7,9,10	China
Ng *et al*.^25^	67 vs. 67/69^b^	60	LCS	55 vs. 110	20	EA	Sham-EA	1,3,4,5,6,7,11	China
Ou *et al*.^35^	63 vs. 64	60.7	LCS	28 vs. 28	20	EA	Standard care	1,2,5,7,11	China
Shen *et al*.^27^	60/59 vs. 60^b^	64.6	LCS; OCS	62 vs. 29	30	EA	Standard care	1,4,7,8	China
Wang ^29^	56 vs. 55	51.4	LCS; OCS	35 vs. 35	30	EA	Standard care	1,2,3,7,8,11	China
Wang *et al*.^28^	52/51/49 vs. 51^c^	50	OCS	58 vs. 20	30	EA	Standard care	1,2,4,11	China
Wang ^30^	65/62/62 vs. 64^c^	51.3	OCS	60 vs. 20	20	EA	Standard care	1,2,3	China
Wang *et al*.^31^	60 vs. 60	61.4	LCS	125 vs. 123	30	EA	Sham-EA	1,2,4,5,6,7,10,11	China
Wei *et al*.^32^	NA^b^	NA	NA	85 vs. 50	30	EA	Standard care	1,2,5	China
Yang *et al*.^33^	62/61 vs. 61^b^	62.9	LCS	70 vs. 35	30	EA	Standard care	1,2,4,5,6,7,11	China
Zhang *et al*.^34^	63 vs. 60	57.9	OCS	19 vs. 20	30	EA	Sham-EA	1,2,3,5,11	China

a Present as intervention versus control groups.

b Triple-arm study design.

c Four-arm study design.

Outcome 1. Time to first flatus. 2. Time to first defecation. 3. Bowel sound recovery time. 4. Time tolerability of liquid, semiliquid, solid food. 5. Length of postoperative hospital stay, day. 6. Time to first ambulation, day. 7. Pain score. 8. Vomiting score. 9. Nausea score. 10. Abdominal distension score after operation. 11. Number of patients with complications.

EA, Electroacupuncture; ERS, Enhanced recovery after surgery; LCS, laparoscopic colorectal surgery; NA, not available; OCS, open colorectal surgery.

Table 2 provides further details of the acupoints used in the included studies. Among the 16 studies, the majority (i.e. 13 studies) utilized acupoint ST36 either alone or in combination with other acupoints (such as ST39) for alleviating POI^16,20,21,23,25,27–29,31–35^. Three studies used a variety of acupoints^22,24,30^. These acupoints include LU7, LU9, SJ6, GB34, LI9, ST39, CV12, and ST25^22,24,30^. In terms of the waveforms of EA, seven trials utilized continuous waveforms^20–24,27,32^, whereas two trials utilized dilatational waveforms characterized by pulsating or intermittent patterns of electrical current^29,33^. However, seven trials did not provide information on the waveform^16,25,28,30,31,34,35^. Regarding the timing of EA administration, four studies performed EA before the surgical operation^21,23,27,28^, while EA was administered after the operation in 12 trials^16,20,22,24,25,29–35^.

**Table 2:** Details of acupoint (*n*=16).

Author (year)	Acupoint	Waveforms	Electrical frequency	Needle Retention time (min)
Deng *et al*.^16^	ST36, ST37, JM8	NA	NA	30^a^
Gong *et al*.^20^	ST36	Continuous	NA	30^a^
Li *et al*.^21^	ST36, SP6, PC6, GV20	Continuous	2/100 Hz	30^b^
Long *et al*.^22^	LU7, LU9	Continuous	2 Hz, 1–2 mA	30^a^
Mai *et al*.^23^	ST36, ST37, ST39	Continuous	2 Hz, 2–3 mA	30^b^
Meng *et al*.^24^	SJ6, GB34	Continuous	2 Hz	20^a^
Ng *et al*.^25^	ST36, SP6, LI4, TE6	NA	100Hz	20^a^
Ou *et al*.^35^	ST 36	NA	NA	20^a^
Shen *et al*.^27^	ST36, RN16, RN17, PC6	Continuous	2/100 Hz, 2–3 mA	30^b^
Wang ^29^	ST36	Dilatational	NA	30^a^
Wang *et al*.^28^	ST36, ST25, ST37, ST39, CV12, PC6	NA	2 Hz, 2–3 mA	30^b^
Wang ^30^	LI9, ST39, CV12, ST25	NA	NA	20^b^
Wang *et al*.^31^	ST36, ST37, ST25, RN12	NA	100Hz	30^a^
Wei *et al*.^32^	ST36, ST37, ST39, CV6, CV8, LI4, PC6	Continuous	50–100Hz	30^a^
Yang *et al*.^33^	ST36, ST25	Dilatational	2/100Hz	30^a^
Zhang *et al*.^34^	ST36	NA	2 Hz	30^a^

a Postoperation.

b Before operation.

NA, not available.

### Risk of bias assessment

**Figure 2:**
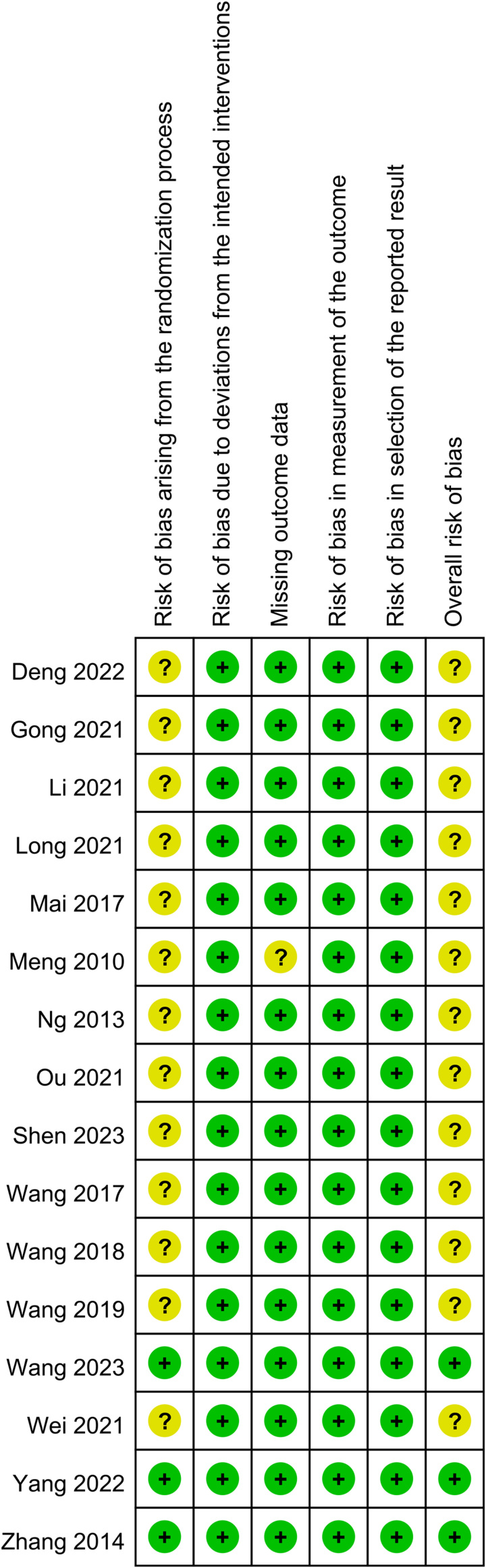
The risks of bias of individual studies.

Figure 2 summarizes the results of the risk of bias assessment. Of the 16 studies, 13 were categorized as ‘having concernsʼ over the risk of bias associated with the randomization process^16,20–25,27–30,32,35^. For the risk of bias related to missing outcome data, one study was considered to raise ʻsome concernsʼ^24^. The assessment of the overall risk of bias across all studies revealed that ʻsome concernsʼ existed in 13 studies^16,20–25,27–30,32,35^, indicating the presence of certain methodological limitations or concerns that could impact the reliability and validity of the findings. In contrast, the overall risk of bias was considered low in three studies^31,33,34^.

### Primary and secondary outcomes

#### Primary outcome: times to first flatus, first defecation, and bowel sound recovery

**Figure 3:**
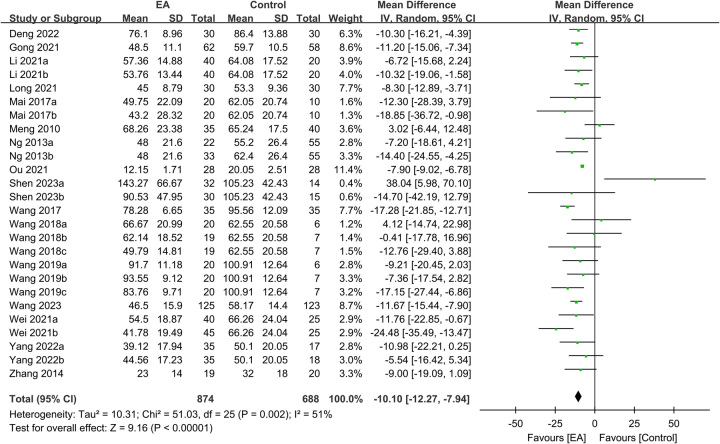
Forest plot showing the association of electroacupuncture (EA) use with the time to first flatus (*n*=1562). IV, inverse variance; CI, confidence interval; SD, standard deviation.

**Figure 4:**
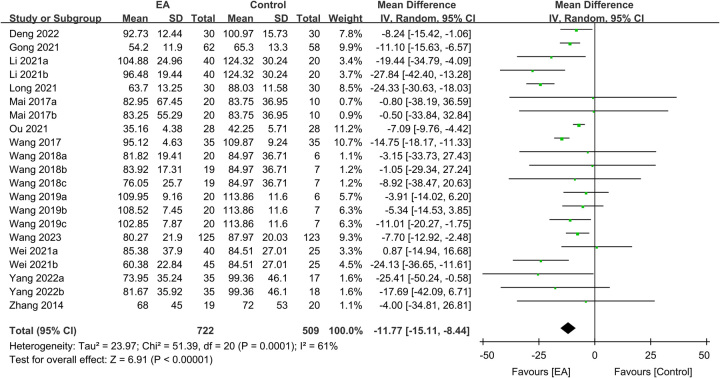
Forest plot demonstrating the correlation between electroacupuncture (EA) use and the time to first defecation (h). IV, inverse variance; CI, confidence interval; SD, standard deviation.

**Figure 5:**
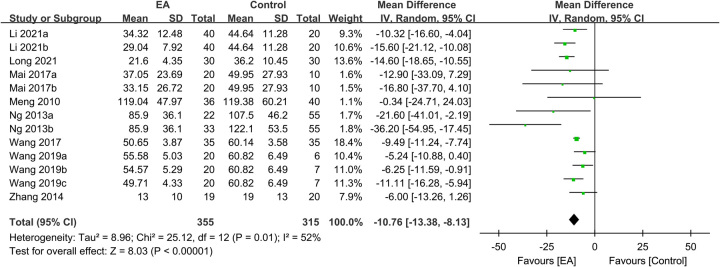
Forest plot showing the association of electroacupuncture (EA) use with bowel sound recovery time (h). IV, inverse variance; CI, confidence interval; SD, standard deviation.

Regarding TFF, the analysis included data from 1562 participants, with 874 in the EA group and 688 in the control group. The meta-analysis revealed a significantly shorter TFF in patients receiving EA than in those receiving standard care (MD: −10.1 h, 95% CI: −12.27–−7.94, *P*<0.00001, *I*^2^=51%) (Fig. 3)^16,20–25,27–35^. The sensitivity analysis showed consistent findings. Furthermore, an analysis of 1231 participants demonstrated a shorter TFD in the EA group than in the control group (MD: −11.77 h, 95% CI: −15.11–−8.44, *P*<0.00001, *I*^2^=61%, sensitivity analysis: consistent) (Fig. 4)^16,20–23,28–35^. Additionally, pooled results from 670 patients showed a shorter TBSR in the EA group than in the control group (MD: −10.76 h, 95% CI: −13.38–−8.13, *P*<0.00001, *I*^2^=52%, sensitivity analysis: consistent) (Fig. 5)^21–25,29,30,34^. An inspection of the funnel plot suggested a low risk of publication bias for these three outcomes (Supplemental Figs. 1–3, Supplemental Digital Content 1, http://links.lww.com/JS9/B232), indicating the satisfactory reliability of the results derived from the included studies.

#### Secondary outcomes: impact of EA on postoperative pain

**Figure 6:**
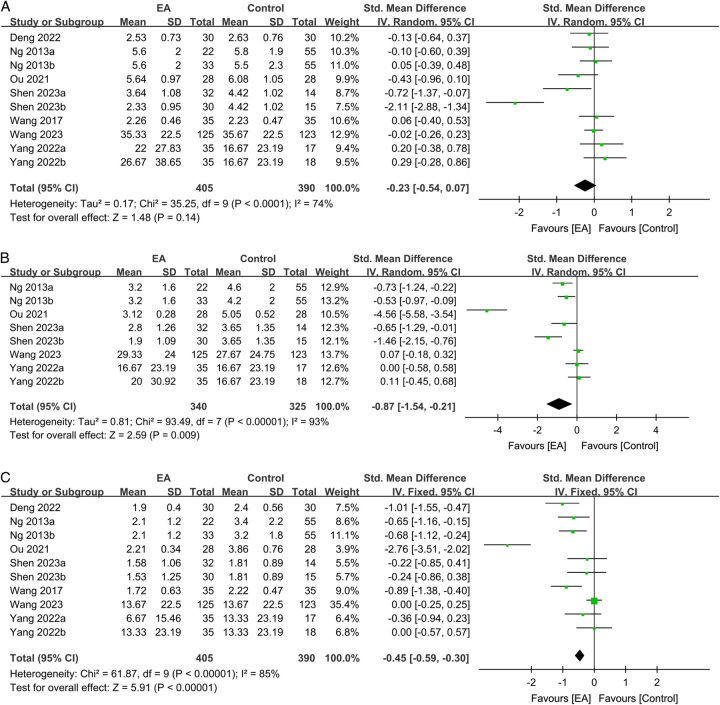
Forest plot showing the correlations of electroacupuncture (EA) use with pain score on (A) day 1; (B) day 2; and (C) day 3. IV, inverse variance; CI, confidence interval; Std, standardized.

Figure 6 presents the effect of EA on the severity of pain from postoperative day one to day three. On postoperative day one, albeit not statistically significant, there was a trend indicating an association between EA use and less severe postoperative pain (SMD: −0.23, 95% CI: −0.54–0.07, *P*=0.14, *I*^2^=74%, *n*=795 patients, sensitivity analysis: consistent) (Fig. 6A)^16,25,27,29,31,33,35^. On postoperative day two, patients in the EA group experienced significantly less severe pain than those in the control group (SMD: −0.87, 95% CI: −1.54–−0.21, *P*=0.009, *I*^2^=93%, *n*=655 patients, sensitivity analysis: consistent) (Fig. 6B)^25,27,31,33,35^. Similarly, on postoperative day three, the severity of pain in patients undergoing EA was lower than that in those receiving standard care (SMD: −0.45, 95% CI: −0.59–−0.3, *P*<0.00001, *I*^2^=85%, *n*=795 patients, sensitivity analysis: consistent) (Fig. 6C)^16,25,27,29,31,33,35^. A low risk of publication bias for these outcomes (Supplemental Figs. 4–5, Supplemental Digital Content 1, http://links.lww.com/JS9/B232) suggested that the included studies in the current meta-analysis were representative.

#### Secondary outcomes: impact of EA on ambulation and food intake

**Figure 7:**
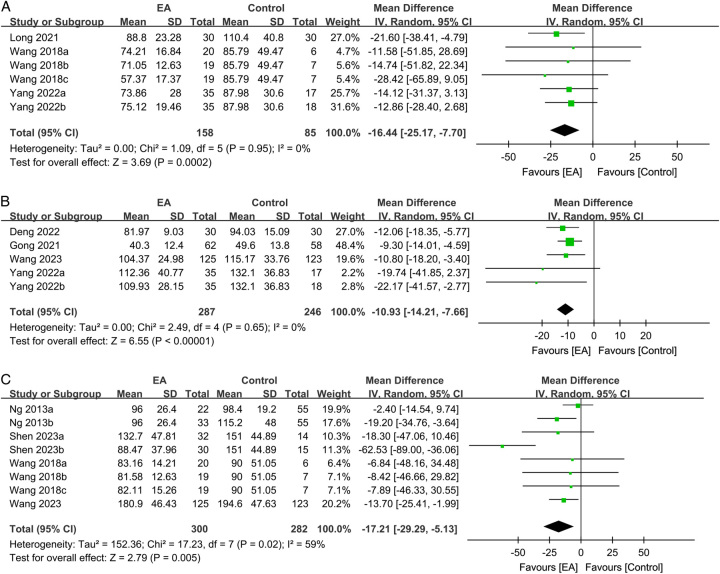
Forest plot showing the associations of electroacupuncture (EA) use with the time to tolerability of (A) liquid; (B) semiliquid; and (C) solid food. CI, confidence interval; IV, inverse variance; SD, standard deviation.

The time to first ambulation did not differ significantly between the groups receiving EA and standard care (MD: −5.9 h, 95% CI: −12.34–0.55, *P*=0.07, *I*^2^=52%, *n*=518 patients, sensitivity analysis: consistent)^25,31,33^. Regarding the impact of EA on postoperative liquid/food intake, the use of EA was associated with a significant reduction in the time to tolerability of liquid (MD: −16.44 h, 95% CI: −25.17–−7.7, *P*=0.0002, *I*^2^=0%, *n*=243 patients) (Fig. 7A)^22,28,33^. However, the sensitivity analysis showed inconsistent findings when the study by Long *et al*.^22^ was excluded. With respect to the time to tolerability of semiliquid food, EA revealed a significant benefit (MD: −10.93 h, 95% CI: −14.21–−7.66, *P*<0.00001, *I*^2^=0%, *n*=533 patients, sensitivity analysis: consistent) (Fig. 7B)^16,20,31,33^. Furthermore, the use of EA resulted in a shorter time to tolerability of solid food (MD: −17.21 h, 95% CI: −29.29–−5.13, *P*=0.005, *I*^2^=59%, *n*=582 patients, sensitivity analysis: consistent) (Fig. 7C)^25,27,28,31^.

#### Secondary outcomes: impact of EA on postoperative complications and hospital LOS

**Figure 8:**
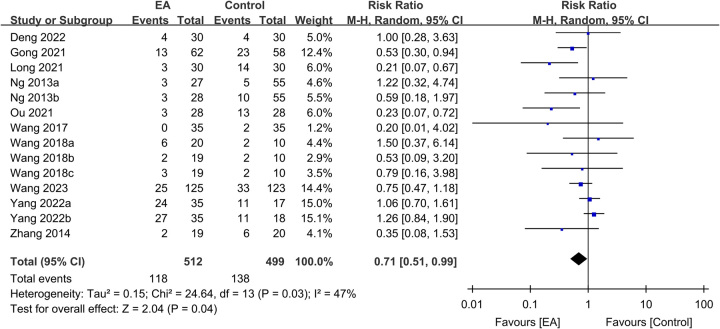
Forest plot showing the association between electroacupuncture (EA) use and the risks of postoperative complications. CI, confidence interval; IV, inverse variance; SD, standard deviation.

**Figure 9:**
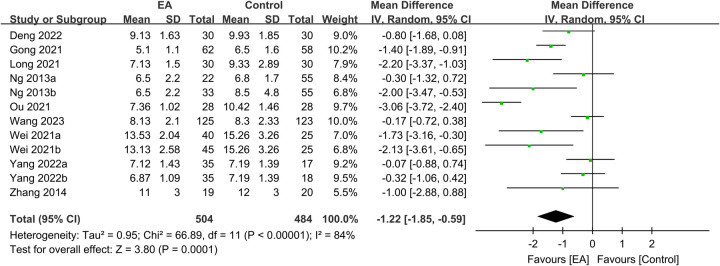
Forest plot showing the association of electroacupuncture (EA) use with length of postoperative hospital stay (days). CI, confidence interval; IV, inverse variance; SD, standard deviation.

The use of EA was linked to a lower risk of overall complications than that in the controls (RR: 0.71, 95% CI: 0.51–0.99, *P*=0.04, *I*^2^=47%, *n*=1011 patients, sensitivity analysis: inconsistent) (Fig. 8)^16,20,22,25,28,29,31,33–35^. Pooled results based on 988 participants revealed a shorter hospital LOS in the EA group than in the control group (MD: −1.22 days, 95% CI: −1.85–−0.59, *P*=0.0001, *I*^2^=84%, *n*=988 patients, sensitivity analysis: consistent) (Fig. 9)^16,20,22,25,31–35^. A low risk of publication bias for these outcomes (Supplemental Figs. 6–7, Supplemental Digital Content 1, http://links.lww.com/JS9/B232) suggested that the included studies in the current meta-analysis were representative.

### Subgroup analysis of the primary outcome

Table 3 summarizes the findings of the subgroup analysis. Subgroup analysis based on the type of surgery (laparoscopy vs. open surgery), type of control group (standard care vs. sham EA), position of acupoint (ST36 vs. other acupoints), and time of EA (postoperative vs. preoperative) demonstrated no influence of these variables on the primary outcome (TFF).

**Table 3:** Subgroup analysis on primary outcome.

Outcome or subgroup	Data sets	Participants (*N*)	Effect estimate; MD [95% CI]	*I*^2^
Type of surgery				
Laparoscopy	10	874	−8.52 [−9.52–−7.53]	0%
Open surgery	9	257	−10.02 [−14.34–−5.70]	0%
Type of control group				
Standard care	20	990	−10.00 [−12.79–−7.20]	60%
Sham-EA	6	572	−10.75 [−13.60–−7.90]	0%
Position of acupoint				
ST36-based	21	1347	−10.73 [−13.19–−8.27]	53%
Other acupoint	5	215	−7.67 [−13.28–−2.06]	52%
Time of EA				
Postoperative	17	1213	−10.60 [−12.91–−8.29]	58%
Preoperative	9	349	−7.01 [−13.61–−0.41]	38%

EA, electroacupuncture; MD, mean difference.

### Certainty of evidence

Supplemental Table 1 (Supplemental Digital Content 1, http://links.lww.com/JS9/B232) summarizes the certainty of evidence across different outcomes. A high level of certainty was assigned to four outcomes (TFF, TBSR, time to tolerability of liquid, and time to tolerability of semiliquid food), indicating their robustness and reliability. Four other outcomes had a moderate level of certainty (i.e. TFD, time to first ambulation, time to tolerability of solid food, and postoperative complications), whereas the other four outcomes (i.e. postoperative pain on days 1, 2, and 3, as well as hospital LOS) had a low level of certainty.

## Discussion

The current meta-analysis, focusing on 16 studies with a total of 1562 patients demonstrated several positive outcomes associated with the use of EA in patients undergoing colorectal surgeries. First, patients who received EA had shorter gastrointestinal functional recovery times, including TFF, TFD, and TBSR, than those who received standard care. Consistently, patients undergoing EA showed shorter times to tolerability of liquid, semiliquid, and solid food intake. Despite the lack of difference in time to first ambulation between the EA and control groups, the former exhibited a trend of reduced postoperative pain on day one and experienced significantly less severe pain on days two and three compared to the controls. Furthermore, our results demonstrated the association of EA with a lower risk of overall complications and a shorter hospital LOS. These findings highlight the potential benefits of incorporating EA into perioperative care to enhance recovery.

POI, which is characterized by the temporary cessation of bowel movement, is a prevalent postoperative complication, particularly following open abdominal surgery. Its etiology is believed to be an interplay between sympathetic input, mediator release, the inflammatory cascade, and analgesic impact^36^. Although there is no ideal treatment modality for POI, previous studies have provided compelling evidence supporting the effectiveness of EA in alleviating POI and its associated symptoms^13,14^. EA is a therapeutic approach that involves the insertion of fine needles into acupoints followed by the application of low-level electrical currents to achieve therapeutic outcomes by synergistically combining the stimulatory effects of acupuncture and electrostimulation. Compared with conventional acupuncture, one distinct advantage of EA is its ability to stimulate acupoints with precise modulation of frequency and intensity, thereby allowing for accurate and quantifiable adjustments^25^. Furthermore, EA triggers analgesic effects by activating a range of bioactive substances through peripheral, spinal, and supraspinal mechanisms^37^. Previous investigations have demonstrated an association of acupoints ST36 and SP6 with the cAMP-CREB pathway and the mRNA expression profile in the brainstem of morphine-tolerant mice^38^. Moreover, EA exhibits anti-inflammatory effects by suppressing the expression of adenosine and substance P, which contribute to inflammatory pain^39^. Furthermore, EA activates the cholinergic anti-inflammatory pathway, leading to a reduction in the release of proinflammatory cytokines, including TNF-α, IL-1β, and IL-10, thereby mitigating postoperative inflammatory responses and diminishing the incidence of unexplained fever in patients receiving craniotomy^40^.

One previous meta-analysis, including an article published before 2017, reported a correlation between acupuncture and related therapies with enhanced gastrointestinal recovery after colorectal surgery, as reflected by a shorter TFF (i.e. −15.79 h) compared to the control group^41^. However, the strength of the evidence of this analysis was limited by the inclusion of only four RCTs. Two other meta-analyses, which included patients undergoing various abdominal surgeries, also found a shorter TFF with the use of mixed acupuncture techniques (i.e. EA, TEA, or manual acupuncture) than with the control group. Nevertheless, the significance of their findings was impaired by high heterogeneity (*I*^2^=94% and *I*^2^=90%, respectively)^13,14^ probably due to the inclusion of different acupuncture techniques and various surgical procedures. Additionally, as only a small subset of studies (i.e. three and five RCTs, respectively) in those meta-analyses focused on laparoscopic procedures, their findings^13,14^ may not be applicable to real-world scenarios, considering the increasing popularity of laparoscopic procedures in current clinical practice^42–44^. Our meta-analysis of 1552 patients found a significant association between EA and a shorter TFF (i.e. −10.1 h) compared to standard care. The relatively low heterogeneity (51%) in our analysis may be attributed to the precise application of stabilized electrical stimulation during the EA procedure and the inclusion of patients specifically undergoing colorectal surgery. In addition, a substantial number of studies in our meta-analysis that included patients undergoing laparoscopic colorectal surgery (i.e. nine RCTs) may provide updated information for clinical practice.

Our current meta-analysis revealed a lower pain score on postoperative days two and three in patients who received EA than in the control group, despite only borderline significance on day one. Nevertheless, our finding of a borderline reduction in pain score on postoperative day one aligned with that of a previous study that demonstrated the effectiveness of TEAS for pain alleviation at short-term time points (i.e. 4 h, 12 h, and 24 h) following laparoscopy^12^. Our inclusion of studies that involved both open laparotomy and laparoscopic procedures may have hampered the significance of our findings. Considering the association between the development of POI and aggravation of postoperative pain^45^, our findings of a shorter TFF (i.e. lower risk of POI) in patients receiving EA may partly explain their lower pain scores observed on days two and three than those in the controls.

According to the traditional meridian theory in traditional Chinese medicine, specific anatomical locations along the meridians, known as acupoints, are believed to have a direct influence on internal organs. Zusanli (ST36) is an acupoint located along the stomach meridian of the foot-yangming meridian channel. A previous RCT has demonstrated that not only could EA at ST36 alleviate inflammatory reactions, but it could also attenuate intra-abdominal pressure in septic patients^46^. Similarly, several animal studies have shown the effectiveness of EA in improving gastrointestinal motility^46–48^. These findings, therefore, suggest that EA at ST36 has therapeutic potential for preventing POI and optimizing gastrointestinal function.

This study had several limitations that should be acknowledged. First, the fact that all 16 included RCTs were conducted in China may limit the extrapolation of the findings to other populations of different ethnic and geographic backgrounds, as well as healthcare settings. Second, the wide variation in sample size (i.e. from 19 to 125 in the EA group and from 20 to 123 in the control group) may have impaired the statistical power and precision of the results. Third, although we found a significant positive effect of EA on the alleviation of POI regardless of the choice of acupoints, the majority of studies focused on acupoint ST36 alone or in combination with other acupoints, and only a few studies explored other acupoints. Therefore, the impact of EA on different acupoints cannot be differentiated. Fourth, the difference in the timing of EA administration among the studies (i.e. preoperative or postoperative) may be a potential confounder that could influence the outcomes. Finally, the finding of some concerns in our assessment of the risk of bias in the majority of studies suggested the presence of methodological limitations that may impact the reliability and validity of our results. Furthermore, the extended publication period of the included studies (i.e. from 2013 to 2023) could introduce potential biases associated with changes in clinical practice and advancements in surgical techniques over time.

In conclusion, the current meta-analysis of 16 studies involving 1562 patients demonstrated a positive association between the use of EA and enhanced gastrointestinal recovery (e.g. time to first flatus), shortened time to tolerability of liquid/food, reduced postoperative pain on day two and three, as well as a lower risk of complications and shorter hospital stays compared to patients receiving standard care. Although our findings support the multifaceted advantages of EA in optimizing the recovery trajectory in patients undergoing colorectal surgery, the inclusion of only Asian studies warrants further large-scale investigations to elucidate the benefits of EA in patients with different ethnic and geographical backgrounds.

## Ethical approval

Not applicable.

## Consent

Not applicable.

## Sources of funding

Not applicable.

## Author contribution

H.-T.C. and K.-C.H.: conceptualization, methodology, and software; Y.-T.H. and. J.-Y.W.: data curation; K.-C.H. and I.-W.C.: writing – original draft preparation; C.-H.H. and C.-M.L.: visualization, investigation; C.-K.S.: supervision; K.-C.H. and I.-W.C.: software and validation; K.-C.H. and H.-T.C.: writing – reviewing and editing; I.-W.C. and C.-K.S.: contributed equally as corresponding authors to this study; H.-T.C. and K.-C.H.: contributed equally as first authors to this study.

## Conflicts of interest disclosure

The authors declare that they have no financial conflicts of interest with regard to the content of this report.

## Research registration unique identifying number (UIN)

1. Name of the registry: PROSPERO.
2. Unique identifying number or registration ID: CRD42023433310.
3. Hyperlink to your specific registration (must be publicly accessible and will be checked): https://www.crd.york.ac.uk/prospero/display_record.php?ID=CRD42023433310.

## Guarantor

Hsiao-Tien Chen, Kuo-Chuan Hung, I-Wen Chen, and Cheuk-Kwan Sun.

## Data availability statement

The datasets used and/or analyzed in the current study are available from the corresponding author upon reasonable request.

## Provenance and peer review

Not commissioned, externally peer-reviewed.

## Supplementary Material

**Figure:**
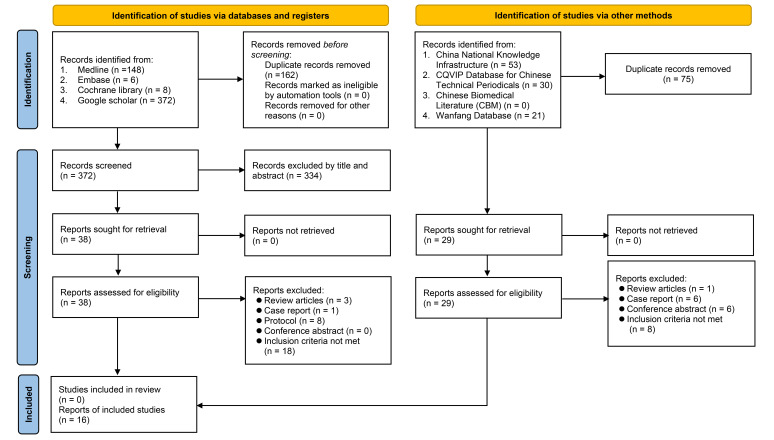

