# Supplementary material for: Efficacy of electroacupuncture in improving postoperative ileus in patients receiving colorectal surgery: a systematic review and meta-analysis
Source: Int J Surg. 2023 Nov 2;110(2):1113–25. doi: 10.1097/JS9.0000000000000848 (PMC10871621; doi:10.1097/JS9.0000000000000848)

**Supplemental table 1.** Summary of findings for the main comparison

| Outcomes | Relative effect (95% CI) | N | Certainty of the evidence (GRADE) | Comments |
| --- | --- | --- | --- | --- |
|  |  |  |  |  |
| 1. Time to first flatus | -10.10 [-12.27, -7.94] | 1562 | ⨁⨁⨁⨁ High | - |
| 1. Time to first defecation | -11.77 [-15.11, -8.44] | 1231 | ⨁⨁⨁◯ moderate | b |
| 1. Time to bowel sound recovery | -10.76 [-13.38, -8.13] | 670 | ⨁⨁⨁⨁ High | - |
| 1. Postoperative pain on day 1 | -0.23 [-0.54, 0.07] | 795 | ⨁⨁◯◯ Low | a, b |
| 1. Postoperative pain on day 2 | -0.87 [-1.54, -0.21] | 665 | ⨁⨁◯◯ Low | a, b |
| 1. Postoperative pain on day 3 | -0.45 [-0.59, -0.30] | 795 | ⨁⨁◯◯ Low | a, b |
| 1. Time to first ambulation | -5.90 [-12.34, 0.55] | 518 | ⨁⨁⨁◯ moderate | b |
| 1. Time to tolerability of liquid | -16.44 [-25.17, -7.70] | 243 | ⨁⨁⨁⨁ High | - |
| 1. Time to tolerability of semiliquid | -10.93 [-14.21, -7.66] | 533 | ⨁⨁⨁⨁ High | - |
| 1. Time to tolerability of solid food | -17.21 [-29.29, -5.13] | 582 | ⨁⨁⨁◯ Moderate | b |
| 1. Postoperative complications | 0.71 [0.51, 0.99] | 1011 | ⨁⨁⨁◯ Moderate | a |
| 1. Hospital length of stay | -1.22 [-1.85, -0.59] | 988 | ⨁⨁◯◯ Low | a, b |

MD: mean difference; SMD: standardized mean difference; RR: risk ratio

Comments:

^a^wide 95% CI; ^b^The I square is more than 50%.

GRADE Working Group grades of evidence:
High certainty: We are very confident that the true effect lies close to that of the estimate of the effect
Moderate certainty: We are moderately confident in the effect estimate: The true effect is likely to be close to the estimate of the effect, but there is a possibility that it is substantially different
Low certainty: Our confidence in the effect estimate is limited: The true effect may be substantially different from the estimate of the effect
Very low certainty: We have very little confidence in the effect estimate: The true effect is likely to be substantially different from the estimate of effect

**Supplemental Figure 1.** Funnel plot showing a low risk of publication bias on time to first flatus


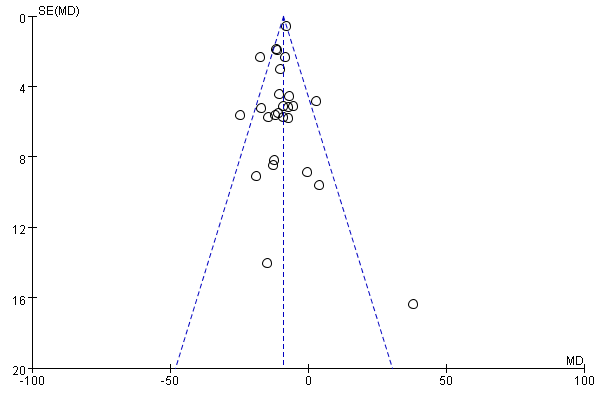


**Supplemental Figure 2.** Funnel plot showing a low risk of publication bias on time to first defecation.


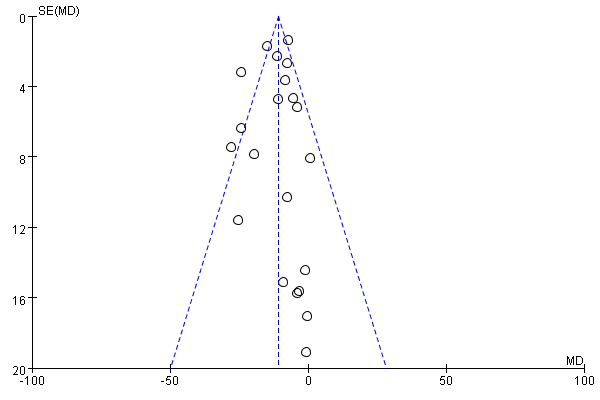


**Supplemental Figure 3.** Funnel plot showing a low risk of bias on bowel sound recovery time.


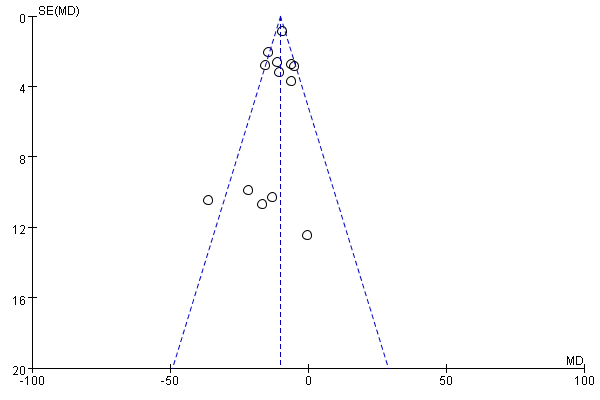


**Supplemental Figure 4.** Funnel plot showing a low risk of bias on pain score at postoperative day one.


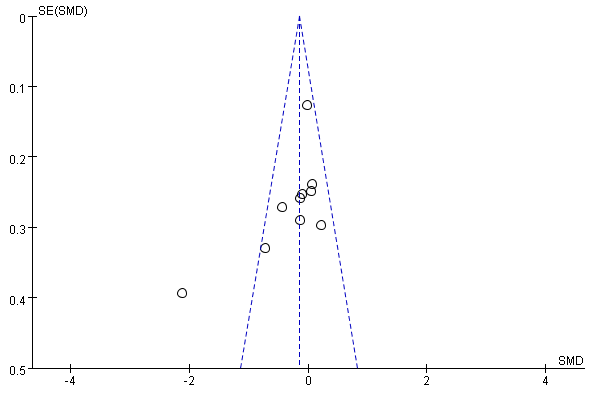


**Supplemental Figure 5.** Funnel plot showing a low risk of bias on pain score at postoperative day three.


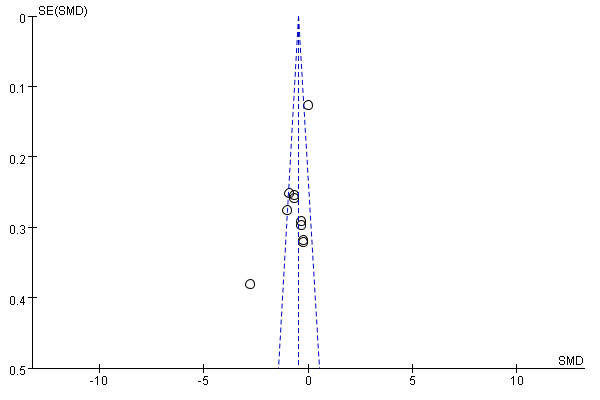


**Supplemental Figure 6.** Funnel plot showing a low risk of bias on overall complications.


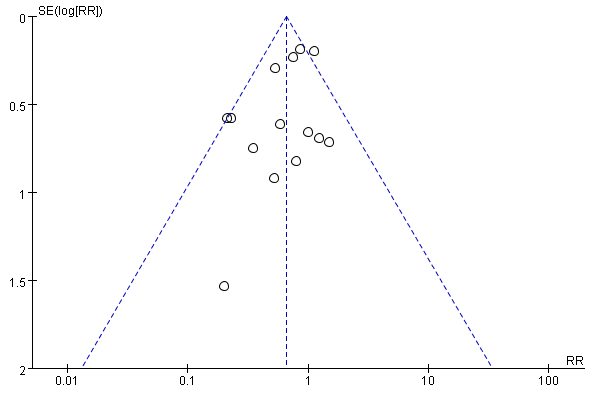


**Supplemental Figure 7.** Funnel plot showing a low risk of bias on hospital length of stay.


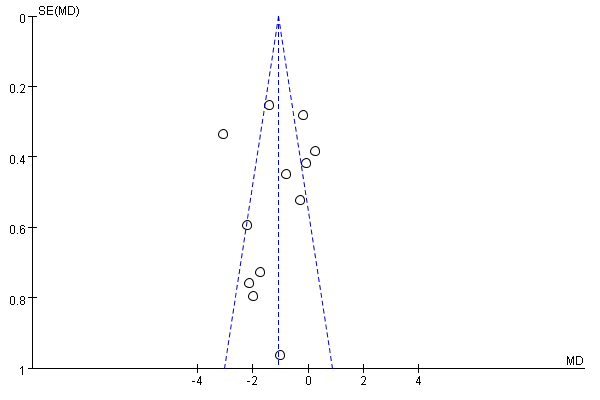

Supplement: Supplementary file 1 [file js9-110-1113-s001.docx]
